# Development of an implementation intention-based intervention to change children’s and parent-carers’ behaviour

**DOI:** 10.1186/s40814-017-0171-6

**Published:** 2017-07-17

**Authors:** Karen Davies, Christopher J. Armitage, Yin-Ling Lin, James Munro, Tanya Walsh, Peter Callery

**Affiliations:** 1 Division of Nursing, Midwifery and Social Work, School of Health Sciences, Jean McFarlane Building, Oxford Road, Manchester, M13 9PL UK; 2 Manchester Centre for Health Psychology, Division of Psychology and Mental Health, School of Health Sciences, Manchester Academic Health Science Centre, Coupland Street, Manchester, M13 9PL UK; 3 Division of Dentistry, School of Medicine and Dentistry, The University of Manchester, JR Moore Building, Oxford Road, Manchester, M13 9PL UK; 4 Division of Nursing, Midwifery and Social Work, School of Health Sciences, University of Manchester, Jean McFarlane Building, Oxford Road, Manchester, M13 9PL UK

**Keywords:** Implementation intentions, Children’s oral health, Animation tutorial, Cleft palate

## Abstract

**Background:**

Implementation intentions enable individuals to translate good intentions into action. Parents and children can find maintaining oral health difficult, as evidenced by the presence of tooth decay. This is a common condition in children in spite of being preventable through the use of regular tooth brushing, fluoride protection and avoiding sugar intake. Even when parents and children are positive about looking after the teeth, they can face challenges in maintaining consistent habits. The aim of this paper is to describe the design of a video animation to teach parents and children how to use implementation intentions to establish new habits to improve oral health, applied in this case, to parents and children with cleft lip and/or palate (CLP).

**Methods:**

Evidence from a qualitative study of parents’ and children’s knowledge, beliefs and behaviour informed the design of an animation forming part of an intervention for children and parents using implementation intentions. The user views generated a set of guiding principles to determine the style and content of a teaching video, whilst an animation designer translated the key messages of implementation intention into images and characters appealing and meaningful to the target audience of children and parents.

**Results:**

A team of researchers, an animation designer and a script writer designed a 2-min video as a teaching tool for children and parents. The team drafted and iteratively refined the content and visuals, with guidance from an advisory group and informal discussions with children in the target age group and their parents. Planning, consulting, designing and production of the animation spanned a total of 20 weeks. The video explains how to formulate ‘if-then’ plans using the voices of a boy and his mother in a conversation, with examples from oral health to illustrate how to enact intentions. It is available via digital media and designed to be delivered by dental care practitioners. The effectiveness of the intervention will be evaluated as part of a feasibility study.

**Conclusion:**

The current study describes the development of an intervention mediated through an animation tutorial that enables children and parents to devise ‘if-then’ plans to improve oral health as a collaborative endeavour between parents and children. The animation uses examples from oral health, but we believe there is scope for exploring application of the intervention to other areas of behaviour.

## Background

This paper presents the rationale and evidence for developing an intervention using implementation intentions as a technique that parents and children can use together to improve children’s oral health. It presents the findings from part of a larger study, ACORN [1], investigating the oral health of children with cleft lip and/or palate (CLP). We use the term ‘parent’ throughout the paper to include anyone with parental responsibilities.

Evidence suggests that there is often a gap between an individual’s intention to achieve a goal and realisation of that goal [2, 3]. Implementation intention, in the form of an ‘if-then’ plan, is an approach that enables people to close the intention-behaviour gap. ‘If-then’ plans are used to enable an individual to articulate how they will achieve specific goals. The ‘if-component’ is a situational cue that prompts a response as part of a ‘then-component’. By using situational cues that occur in an individual’s own context, the desired goal may be easily incorporated in daily routines and become automatic. Although interventions based on implementation intentions have been used to promote behaviour change in various settings, including schools, an implementation intention-based intervention for young children and their parents is a novel adaptation of the approach. Furthermore, using animation as a tutorial to teach parents and children to make ‘if-then’ plans is also a new development. Animation is a potentially powerful means of reaching and engaging children, but little is known about operationalising behaviour change techniques using animation. We identify skills of animation design and script editing to develop an engaging and entertaining video to facilitate children and parents’ use of implementation intentions.

## Methods

### Developmental process for designing the intervention

The paper describes the design of an intervention to improve oral health, employing the MRC framework for development of complex interventions [4]. We designed an implementation intention-based intervention to help individuals to identify constraints on maintaining oral health, agree target behaviour and create a plan of action that could match their specific circumstances. Adopting the MRC framework [4], we identified principles to underpin the design of the intervention from a review of the existing literature and analysis of data from a qualitative study [5].

### Aim of the intervention design

The development phase for the intervention aimed to create an animated tutorial to introduce ‘if-then’ plans to parents and children with CLP to be delivered by dental care practitioners (DCP) to improve oral care.

### Identifying the evidence base

Evidence from the World Health Organization [6] indicates that dental caries (tooth decay) is the most common chronic disease of childhood globally. The UK Children’s Dental Health Survey [7] reported that 31% of 5-year-olds and 46% of 8-year-olds had obvious decay experience in their primary teeth. Untreated decay affecting dentine (a severe form of disease) was found in 28% of 5-year-olds and 39% of 8-year-olds. If the affected teeth cannot be restored, or the pain or infection arising from dental decay becomes too severe, a dentist may refer a child for tooth extraction under general anaesthetic (GA) [8]. Dental caries is the most common hospital diagnosis in children aged between 5 and 9 years, with a substantial number of dental extractions under GA taking place each year. Furthermore, there are socioeconomic disparities in oral health: children from lower income families are more likely to have oral disease than other children of the same age, with well-documented impact on long-term wellbeing [8].

Dental decay is, however, largely preventable through regular tooth brushing, fluoride protection and avoiding free sugars. Maintenance of good oral health is particularly important for children with CLP [9–13]. Decisions about surgery and orthodontic treatment for children with CLP throughout childhood partially depend on adequate oral health. Poor oral health may interfere with healing following a surgical intervention, such as alveolar bone graft [14, 15], or significant dental decay may preclude important orthodontic treatment [16]. Furthermore, early loss of teeth may complicate the orthodontic treatment provided for children with CLP [17].

Policy makers recommend oral health education as a solution for improving children’s oral health, but they provide little detail regarding how practitioners should deliver advice and guidance to children and parents [18, 19]. The lack of effectiveness of interventions focused on improving knowledge to address poor oral health has led to increasing interest in brief behaviour change interventions [19].

The current study [5] utilised a qualitative investigation of parents’ and children’s knowledge, beliefs and attitudes to oral health and the difficulties they experienced in maintaining oral health to inform the development of an intervention delivered through animation. The findings indicated that parents’ attitude towards oral health was mostly positive. They often prioritised their children’s oral health and commented on the importance of encouraging their children to have good oral health behaviour which might be partly stimulated by their experience of their child’s CLP. They not only expressed clear intentions to maintain their child’s oral health but also described difficulties in achieving their intentions. Parents cited frequent challenges in maintaining oral health, influenced by characteristics of their children, such as co-operation, and the context of unpredictable forming considerable obstacles to maintaining consistent habits. Children’s accounts, on the other hand, indicated an intention to be independent in tooth brushing, but they articulated limited knowledge about what was required to maintain oral health. Parents play a key role in enabling children to understand about and maintain oral health [5, 20]. However, the competing demands of family life and social pressure can undermine the parents’ best intentions to prioritise oral health [21]. These tensions may be more apparent for parents of children with CLP where caring for the mouth can be more emotionally laden due to the presence of the cleft [22].

### Identifying/developing appropriate theory

The interview data [5] suggested that parents, and sometimes children, have the motivation to maintain good oral health for those with CLP but face challenges to enact their motivation. Implementation intentions were identified as an appropriate tool to bridge the intention-behaviour gap [2, 3]. Implementation intentions are plans that draw people’s attention to salient cues and ensure that appropriate responses are activated by the cue, helping an individual to achieve a desired goal [2]. Implementation intentions are operationalised as ‘if-then’ plans, where ‘if’ increases the salience of critical cues and ‘then’ prompts automatic activation of specific actions/responses. An essential part of the process is ensuring that individuals articulate the goal that they are trying to achieve as a ‘diagnostic’ stage before creating an ‘if-then’ plan. In the example of oral health, the goal might be brushing teeth twice a day or reducing the frequency of sugar intake. Evidence from other areas of health promotion, such as alcohol reduction and smoking cessation, suggest that people’s intention to change does not necessarily translate into action. Implementation intentions are one means of enabling intentions to be translated into behaviour [3]. Implementation intentions have been proven effective in increasing children’s fruit and vegetable intake [23], preventing uptake of smoking [24], increasing physical activity [25] and reducing adolescents’ alcohol consumption [26]. Moreover, the effects can be long lasting; for example, a study in adolescents showed that the effects of an implementation intention-based intervention aimed at smoking behaviour were sustained 2 years post-intervention [27]. There is some evidence that including a reminder (booster) may not only sustain but also increase initial improvements [27]. There is a growing interest in using implementation intentions in promoting oral health, and studies have evaluated the use of ‘if-then’ plans with adolescents and young adults in oral care [28–31]. Results have been promising, but not conclusive. To date, the technique has not been investigated with young children’s oral health.

### Modelling process and outcomes

Our intervention extends previous work on implementation intention-based interventions in three key respects. First, the intervention extends the concept of forming implementation intentions collaboratively to suit family contexts, by teaching ‘if-then’ plans to parents and children together as a life skill. The qualitative investigation suggested that oral health responsibilities are shared between parents and children with CLP so the intervention should be useable by both children and parents. An audio-visual format minimises the reading ability requirement and was considered a suitable format for children and parents to use together. Furthermore, the interview data from the qualitative study highlighted that the nature of the intention-behaviour gap was highly individualised and influenced by family circumstances. An individualised approach is therefore indicated enabling children and parents to devise their intention implementation plans with specific reference to their own circumstances.

Second, the interviews suggest that oral health can be overshadowed by more immediate or exciting priorities. Previous studies in implementation intentions [32] suggest that the format of delivery does not appear to influence the effectiveness of implementation intention-based interventions. Studies with adults have used both prescribed and self-generated plans, and various modes of delivery, with few differences in achieving behaviour change [33]. Nevertheless, health psychologists are encouraging application of implementation intention through using ‘more engaging planning interventions for specific groups’ (p18) [34]. Parents in our qualitative work often referred to the need for activities to be enjoyable in order to engage children who may have limited interest in oral health care. Some children and parents expressed enthusiasm for new media approaches, such as apps, video clips and games, suggesting that a visual medium would be familiar and preferable to our target population. Animation offers a medium that readily represents mental constructs in a lively format suitable for viewers with variable literacy skills. Moreover, presenting mental constructs visually is believed to support learning, conveying complex points through combining clarity of message with humour [35].

Third, our ambition was that the implementation intention-based intervention could be delivered via health care professionals rather than researchers in order to mirror a real-world application of an intervention. Collaborative implementation intentions, involving two or more people forming plans and performing a specific behaviour together, may be particularly relevant to the context of children and parents promoting oral health, working with a health professional [36]. Underlying mechanisms, such as greater enjoyment, greater motivation and reduced risk of forgetting, could contribute to improved effectiveness.

The development of the intervention was consistent with the TIDieR guidance [37], summarised in Table 1.

**Table 1:** Intervention development using TIDieR checklist [37]

Brief name of intervention	ACORN 2 improving oral health of children with cleft lip and/or palate
Why (rationale, theory, goal)	Oral health problems are common in childhood but preventable. Poor oral health is more common in children with CLP with potential to disrupt orthodontic and surgical treatments. Interventions to increase knowledge have had limited effect on children’s oral health. Implementation intentions are a well-evidenced technique to promote behaviour change. This study describes the development of an intervention to teach parents and children with CLP to use ‘if-then’ plans to implement their intentions to promote oral health behaviour.
What 1. Materials for intervention and training (access to materials) 2. Procedures (describe activities and support activities)	Video animation, ‘if-then’ planning sheets for intervention groups and standard oral health advice for all groups. Training for dental care practitioners (DCP) to help parents and children learn about ‘if-then’ plans and identify their own plans to addressing oral health difficulties
Who provided (describe expertise, background, specific training)	The intervention is designed for delivery by dental care professionals. Professional animator designed video with input from multidisciplinary study team and study advisory group. Health psychologist provided training for DCP DCP deliver intervention (trained in dental health promotion).
How (modes of delivery, e.g. face to face/individual group)	Video training delivered to children and parents together face to face in the home setting. A video animation tutorial is available for them throughout the study. If-then planning sheet completed by parent and child with support from DCP. Families are encouraged to place somewhere prominent.
Where (types of locations)	Children’s home
When and how much (how often is intervention delivered, duration)	One session learning about if-then plans (duration is subject of research) Booster in the form of email/text or letter with picture of original ‘if/then’ plan.
Tailoring (how will intervention be individualized)	Intervention is designed to be individually tailored as part of consultation between DCP and parent/child.
Modifications (any changes during the study)	Reported throughout the feasibility study
How well 1. Intervention fidelity assessed by 2. Actual adherence	Feasibility study involves evaluation of behaviour, clinical outcomes and perception of technique and interaction with plan. The intervention fidelity will be assessed by photographing the initial ‘if-then’ plan, qualitative exit interviews with participants and the DCP who deliver the intervention.

### Piloting

A health psychologist, with specialist knowledge of implementation intentions, cleft specialists, researchers and service users provided guidance throughout the development of the intervention. Service users and their parents were consulted at every step of the intervention design to ensure the language, and the content of the intervention was appropriate for children and parents.

### Ethics

Ethical approval was gained through the NHS NRES Committee West Midlands Ethics Service (15/WM/03).

## Results

### Co-designing an animation to explain implementation intentions

The principal challenge in developing the intervention was to communicate the principles and procedures of implementation intention to children and parents in a brief, engaging animation. Researchers, an animation designer and a script writer collaborated to design a 2-min video as a tutorial for children and parents. The researchers communicated the essential components of implementation intention to the animation designer, who proposed how these could be presented in an audio-visual form. Engaging the creative input of the designer whilst ensuring that the animation conformed to the requirements for implementation intention required several iterations of the script and visual content. The process was guided by the advisory group and informal discussions with children in the target age group and their parents. Planning, consulting, designing and production of the animation spanned a total of 20 weeks (October 2015 to February 2016).

The planning and production broadly followed four steps (Table 2).

**Table 2:** Steps for making animation

Brief: purpose and content based on previous research and findings from qualitative study	
Step 1	Discussion between animation designer and researchers • Explanation of theory in plain language • Initial suggestions for animating the message • Agreeing the tone for the audience: playfulness and fun
Step 2	Finding the key images to carry the message. This was informed by consultation with children and parents and findings from qualitative study. The animation designer identified the following: • Stand out words to be visualised as characters to represent ‘if’ and ‘then’ as a double act to carry the message • Ideas for characters in the narrative (children/adults)
Step 3	Preparing the script: prepared by researchers as a conversation between a parent and child, building on initial suggestions of animation designer.
Step 4	Creating the storyboard: initial drawings prepared for storyboard of the animation based on key images in the script that carried the message well. Script modified by scriptwriter to refine the message. The animation designer prepared animation using stop frame animation. Recording voiceover/adding sound effects and music.

**Fig. 1:**
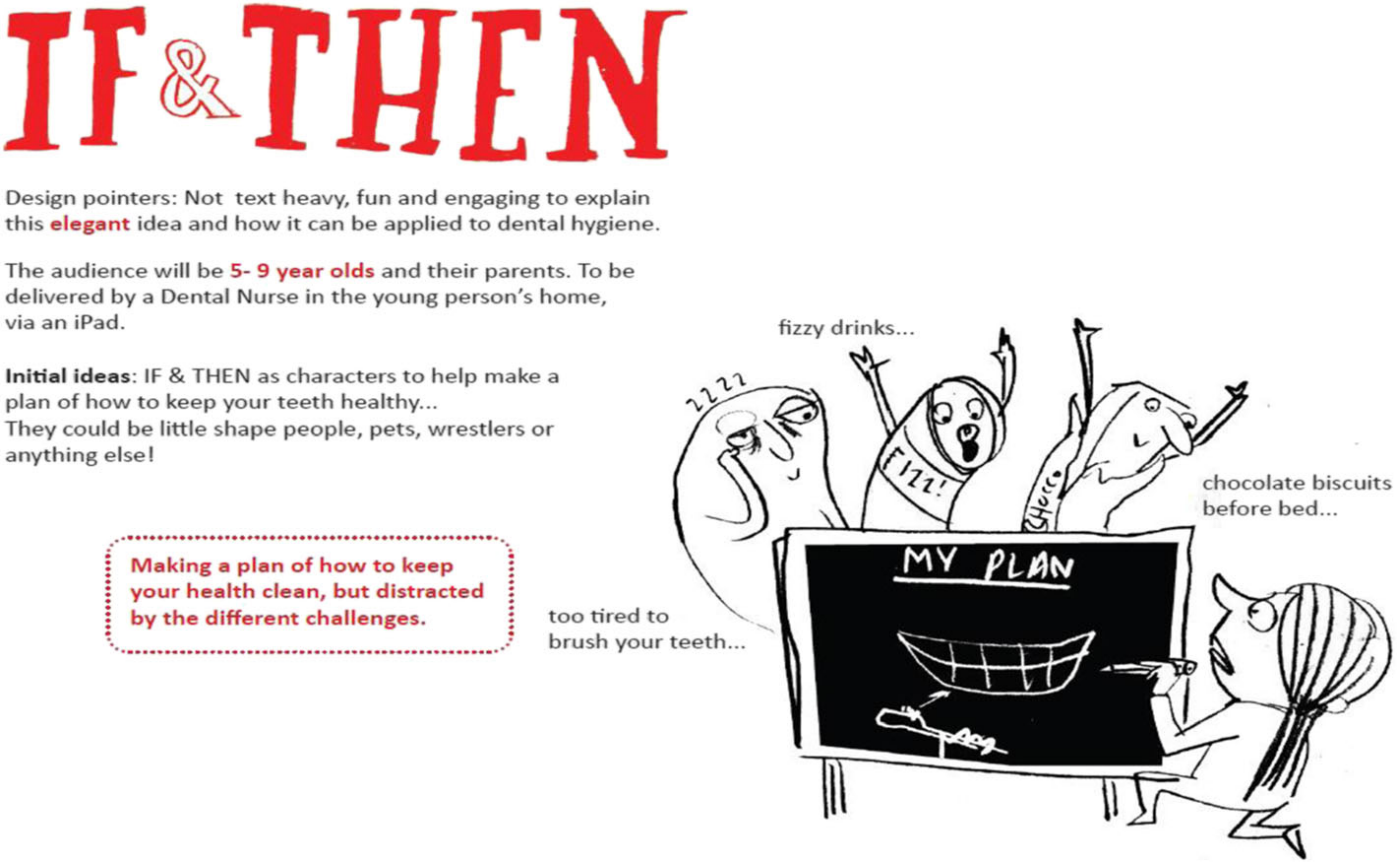
Summary of brief for intervention animation

The process began with a meeting between academic researchers and the animation designer discussing the message and tone of the presentation. This specified that the animation should (i) explain ‘if-then’ plans (what they are, why we use them and how to write one); (ii) engage parents and children and (iii) convey the message in 2 min or fewer. The animation designer then summarised the brief for the presentation (Fig. 1). Figure 1 illustrates the work in development.

**Fig. 2:**
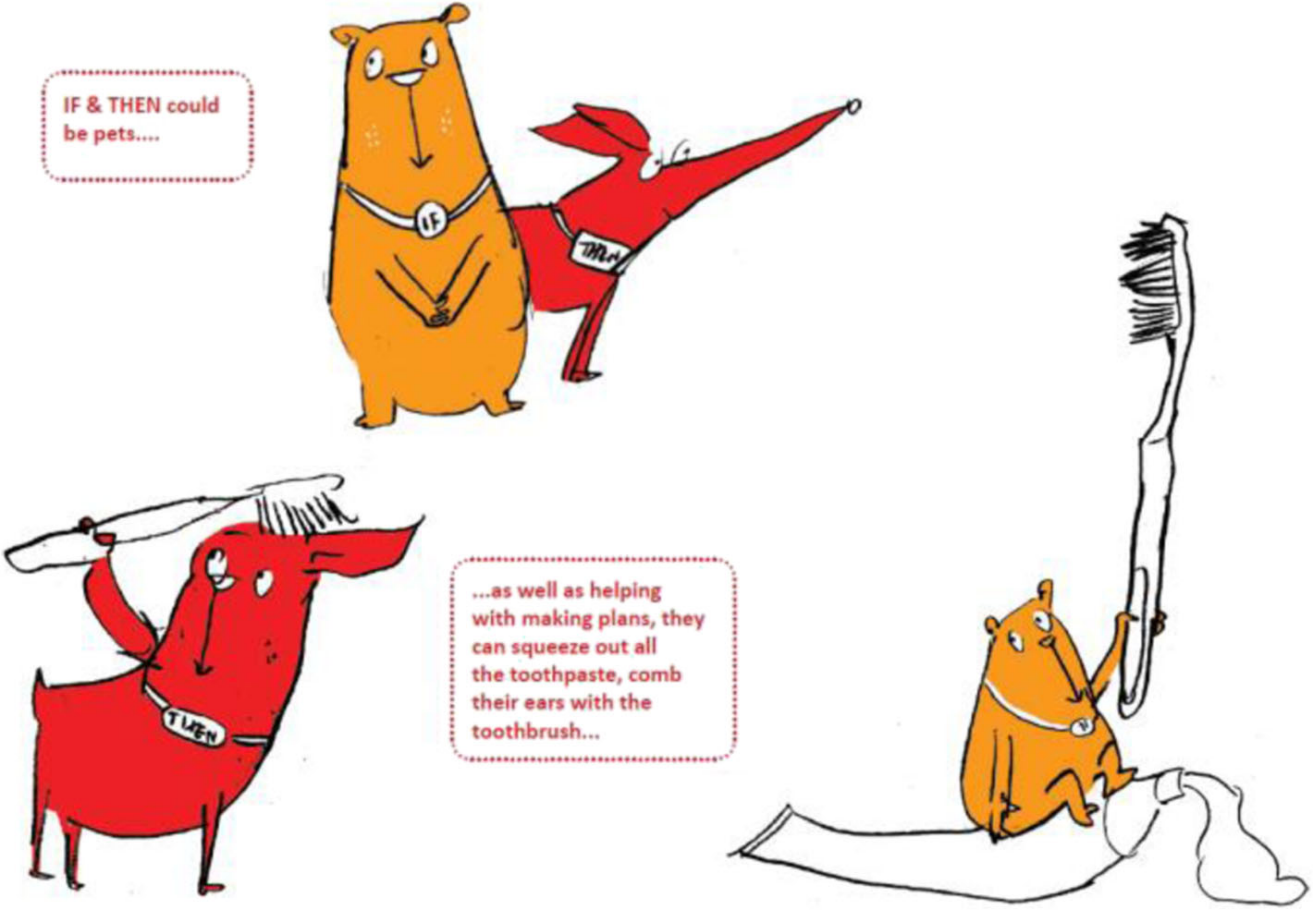
Initial design of characters

During step 2, the animator focused on finding key images and characters to carry the message. This included identifying stand out words (‘if’ and ‘then’) that could be translated into characters who acted mischievously whilst portraying a serious message through the playfulness of a double act. The animation designer explored ideas for the characters, using reflection, sketching and revision (Fig. 2), ‘getting the right personality for an animation character takes a lot of doodling and exploring different ideas’ (JM^1^). Initially, the animation designer offered two alternative visualisations of ‘If’ and ‘Then’ as a pair of wrestlers and two mischievous pets. At a meeting to consult with children and parents, the two pets representing ‘If’ and ‘Then’ were selected as the most appealing to the target audience.

**Fig. 3:**
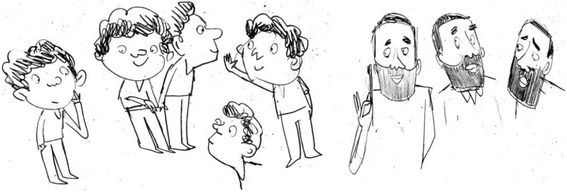
Exploring the personality of the characters

Developing the animation required a creative response from the animation designer to the concept of implementation intentions, illustrated by the comment that ‘it took some sketching and consultation before we hit on the two pets’ (JM) (Fig. 3).

The selection of the gender of the human characters was based on findings from the qualitative study that suggested parents found it more difficult to persuade boys to take care of their teeth and that both mothers and fathers took responsibility for helping their children with tooth brushing. Therefore, the main character was represented by a male child and the adults included both a male and female parent.

In step 3, researchers prepared a draft narrative and script to portray the idea of children and parents making ‘if-then’ plans to manage challenges in looking after the teeth. The interaction between the characters provided a simple and engaging format to deliver information about making an ‘if-then’ plan using a question and answer format that prompted a conversational dialogue involving an explanation of how to make ‘if-then’ plans. A professional scriptwriter refined the script to ensure the animation was succinct and clear, with natural dialogue that complemented rather than duplicated the visual messages, using skills of writing naturalistic dialogue. Professional voiceovers were prepared with a child’s voice as the lead along with an adult female. The child’s voice conveyed enthusiasm and mischief, whilst the adult voice was more measured to represent a parental perspective.

**Fig. 4:**
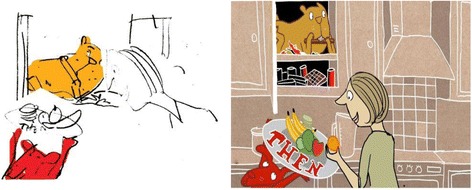
From the initial sketch to the screen shot from animation

**Fig. 5:**
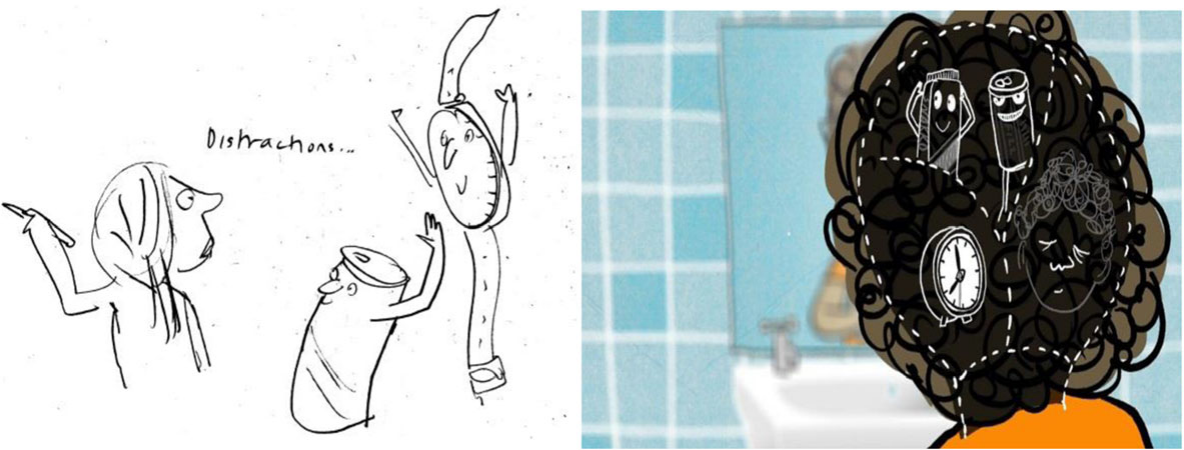
From the initial sketch of distractions to the screen shot from animation

The animation designer prepared the visual storyboard in step 4, breaking the animation into chunks to show the main images. The action was contained in a few scenes in a home setting that was simple to portray visually. The animation then brought life to the characters. The animation designer included occasional ‘fanciful’ frames to develop the humorous content, including a child unicycling over a crocodile infested river, together with sound effects that appeal to younger viewers. Figures 4 and 5 show the development of the video frame from initial sketches.

### Exploiting the medium of animation

The animation designer contributed important features to engage children and parents with the concept of implementation intention plans. ‘If-then’ plans are explained through interaction between imaginary characters making ‘information really easy to absorb- [people are] busy watching the characters’ (JM) and allowing a script to include direct instructions as part of a conversation that appears non-directive to the viewer. Careful attention to the language and pacing of the narrative and images ensures the purpose of the animation is clear. Abstract ideas are explained by simply using key elements of the message (the animator commented that ‘you[researchers] had done a lot of the work to simplify the ideas’) and a plan of explanation that could be translated into messages delivered by the characters (‘everything I did came from the script which you developed yourself’). Iterations of characters, narrative, content and visuals were shown to young children and parents to check the acceptability of the story (e.g. to single parents as well as couples) and the comprehensibility of the messages.

### Implementing the intervention

The intervention has been designed so that any professional providing oral health advice and education can deliver it to parents and children. The intervention is self-explanatory, is easy to use through Youtube Internet video streaming service and requires minimal training. Moreover, the animation as a tutorial for understanding how to formulate ‘if-then’ plans and may be applied more widely to other areas of health behaviour, such as managing diet or exercise.

## Discussion

This paper describes the development of an oral health intervention for children with CLP and their parents. The intervention aims to teach children and parents how to use the technique of implementation intentions to modify oral health behaviour. We developed a short animation, accessible via digital media (Youtube), to convey the key steps in identifying target goals and salient cues to prompt appropriate responses for forming ‘if-then’ plans. The video featured animated characters called ‘If’ and ‘Then’, using a home setting and examples from oral health.

The study illustrates how a relatively abstract concept of implementation intentions has been transformed into an engaging animation format suitable for children and parents. The animation needed to convey a clear message to enable them to understand the technique of ‘if-then’ plans and encourage their engagement. The process of developing the intervention depended on utilising creative skills in visual communication and used an iterative process of co-production between researchers, practitioners, service users and a professional animation designer.

The key messages for developing an engaging and theoretically based animation include, first, involving an appropriately skilled designer in a process of co-production with researchers and clinicians. The animation designer converted abstract ideas relating to behaviour change into visual characters and scenes. In our example, the animation designer suggested a double act of two friendly creatures to represent the ‘if’ and ‘then’ of implementation intentions. Similarly, the script writer created a conversational exchange that complemented the visuals and was amended to match the target audience. Second, qualitative interviews with members of the target audience provided real-life examples to include in the script. This provided a level of confidence that the intervention would be meaningful within children’s and parents’ routine experience. Third, ongoing, consultation with members of the target audience about iterations of the intervention helped identify preferences between alternatives (e.g. different characters), assurance of acceptability (e.g. story lines), clarity of the message and attractiveness of the visuals.

Finally, developing an animation for intervention involved initiating a production partnership between the animation designer, researchers, practitioners and service users. Communication between academic, professional and creative disciplines is key. Design professionals need a clear brief about the concepts underlying an intervention (e.g. implementation intentions) and the target audience (e.g. children with CLP and their parents).

The animation described in this paper forms part of an intervention currently being piloted for use in a randomised feasibility study of using implementation intentions with children (age 5–9 years) and their parents in three centres in the UK during 2016/17 (ISRCTN register DOI 10.1186/ISRCTN45791053) [1]. Parents and children with CLP will be allocated at random into control, intervention and intervention plus booster groups. This study will establish the feasibility of a full randomised control trial to evaluate the effectiveness of the intervention.

The feasibility study aims to evaluate the effectiveness of the intervention in improving oral health behaviours of children with CLP in the home setting as an exemplar of everyday health care behaviour.

Implementation intention is a well-evidenced technique that supports behaviour change [35]. Our adaptation of the approach to help parents and children learn a technique they could apply to their own circumstances is novel. Although it was designed for children with cleft palate, the final version of the animation is not specific and so has potential to support behaviour change in children with other conditions.

## Conclusion

The current study describes the development of an intervention that enables children and parents to devise ‘if-then’ plans to improve oral health as a collaborative endeavour. We used a video-streamed animation for enabling learning about implementation intentions. The animation uses examples from oral health, but we believe there is scope for exploring application of the intervention to other areas of behaviour.

## Abbreviations

ACORN: Improving oral health in cleft lip and/or palate: a health promotion intervention for children and parents
CLP: Cleft lip and/or palate
DCP: Dental care practitioners

## Footnotes

1 The animation designer
